# Mapping of immunome, brain imaging, and cognition in an aging cohort at risk for Alzheimer's disease

**DOI:** 10.1002/alz70855_102122

**Published:** 2025-12-23

**Authors:** Wassim Elyaman

**Affiliations:** ^1^ Department of Neurology, Columbia University, New York, NY, USA

## Abstract

**Background:**

Aging is the greatest risk factor for Alzheimer's disease (AD), with immunosenescence—the gradual decline in immune system function with age—playing a critical role in disease susceptibility. As individuals age, brain‐resident microglia and circulating immune cells undergo functional changes that can compromise their ability to respond effectively to emerging pathology. Dysregulated immune responses, driven by immunosenescence, may exacerbate neuroinflammation and accelerate AD progression. Despite growing recognition of the link between aging, immune dysfunction, and neurodegeneration, no interventions specifically target the impact of immunosenescence during the prodromal stage of AD. Profiling immune changes associated with aging and their connection to early AD biomarkers could uncover druggable pathways, offering transformative opportunities to prevent or delay disease onset.

**Method:**

To identify immune mechanisms underpinning preclinical AD, we performed single‐cell RNA, and T cell and B cell receptor sequencing, capturing >439,000 transcriptomes from 205 participants (ages 29–81) in the Offspring Study of Racial and Ethnic Disparities in Alzheimer's Disease. Plasma proteomics data were also obtained from 86 participants. Immune cell phenotypes and gene expression were correlated with AD biomarkers, including plasma proteins, cortical thickness via MRI, cognitive performance, and parental AD diagnosis.

**Result:**

Our analysis revealed distinct immune signatures associated with cortical integrity. B cell proportions positively correlated with left hemisphere cortical thickness, while CD8+ T cell proportions showed an inverse association. Within the CD8+ T cell population, naïve and mucosal‐associated invariant T (MAIT) cells were enriched in individuals with preserved cortical structure, whereas effector memory cells were reduced. Transcriptomic profiling revealed downregulation of cytotoxicity, antigen processing, and antigen presentation pathways in T cells from individuals with greater cortical thickness and better cognitive function. Additionally, we identified an age‐associated decline in T cell receptor (TCR) repertoire diversity and a reduction in MAIT cell proportions, highlighting the impact of immunosenescence on adaptive immune function and its potential link to neurodegenerative risk.

**Conclusion:**

This study highlights the role of aging‐related immunosenescence in shaping neuroimmune interactions linked to Alzheimer's disease risk. These findings underscore age‐driven immune dysregulation as a key contributor to disease susceptibility and suggest potential targets for immune‐based therapies to mitigate progression.

